# Retinal Architecture in *​RGS9*- and *​R9AP*-Associated Retinal Dysfunction (Bradyopsia)

**DOI:** 10.1016/j.ajo.2015.08.032

**Published:** 2015-12

**Authors:** Rupert W. Strauss, Adam M. Dubis, Robert F. Cooper, Rola Ba-Abbad, Anthony T. Moore, Andrew R. Webster, Alfredo Dubra, Joseph Carroll, Michel Michaelides

**Affiliations:** a Moorfields Eye Hospital, London, United Kingdom; b University College London, Institute of Ophthalmology, London, United Kingdom; c Department of Biomedical Engineering, Marquette University, Milwaukee, Wisconsin; d Department of Ophthalmology, University of California San Francisco, San Francisco, California; e Department of Ophthalmology, Medical College of Wisconsin, Milwaukee, Wisconsin; f Department of Biophysics, Medical College of Wisconsin, Milwaukee, Wisconsin; g Department of Cell Biology, Neurobiology and Anatomy, Medical College of Wisconsin, Milwaukee, Wisconsin

## Abstract

**Purpose:**

To characterize photoreceptor structure and mosaic integrity in subjects with *​RGS9-* and *R9AP*-associated retinal dysfunction (bradyopsia) and compare to previous observations in other cone dysfunction disorders such as oligocone trichromacy.

**Design:**

Observational case series.

**Methods:**

setting: Moorfields Eye Hospital (United Kingdom) and Medical College Wisconsin (USA). study population: Six eyes of 3 subjects with disease-causing variants in *​RGS9* or *R9AP*. main outcome measures: Detailed retinal imaging using spectral-domain optical coherence tomography and confocal adaptive-optics scanning light ophthalmoscopy.

**Results:**

Cone density at 100 μm from foveal center ranged from 123 132 cones/mm^2^ to 140 013 cones/mm^2^. Cone density ranged from 30 573 to 34 876 cones/mm^2^ by 600 μm from center and from 15 987 to 16,253 cones/mm^2^ by 1400 μm from center, in keeping with data from normal subjects. Adaptive-optics imaging identified a small, focal hyporeflective lesion at the foveal center in both eyes of the subject with *RGS9*-associated disease, corresponding to a discrete outer retinal defect also observed on spectral-domain optical coherence tomography; however, the photoreceptor mosaic remained intact at all other observed eccentricities.

**Conclusions:**

Bradyopsia and oligocone trichromacy share common clinical symptoms and cannot be discerned on standard clinical findings alone. Adaptive-optics imaging previously demonstrated a sparse mosaic of normal wave-guiding cones remaining at the fovea, with no visible structure outside the central fovea in oligocone trichromacy. In contrast, the subjects presented in this study with molecularly confirmed bradyopsia had a relatively intact and structurally normal photoreceptor mosaic, allowing the distinction between these disorders based on the cellular phenotype and suggesting different pathomechanisms.

The phenomenon of bradyopsia (“slow vision”: OMIM 608415) was first described by Kooijman and associates in 4 patients from 3 unrelated Dutch families.^1^ Disease-causing sequence variants have been identified in either *RGS9* (encoding a GTPase-activating protein) or *R9AP* (encoding its membrane anchor protein), with both playing a critical role in the recovery phase of visual transduction.^2^, ^3^

Bradyopsia (*RGS9*/*R9AP-*associated retinopathy) is characterized by reduced central vision from childhood, with mild photophobia, absence of nystagmus, normal color vision and night vision, and normal fundus appearance. However, patients report slow visual adaptation to changes in illumination, in both dark- and light-adapted states.^3^, ^4^, ^5^ These symptoms are shared with oligocone trichromacy, another cone dysfunction syndrome characterized by normal fundus appearance, normal or near-normal color vision, reduced visual acuity from infancy, and mild photophobia.^5^, ^6^, ^7^ Unlike *RGS9*/*R9AP*-associated retinopathy, the underlying molecular genetic basis of oligocone trichromacy remains uncertain. Oligocone trichromacy and/or a “marked incomplete achromatopsia (ACHM)-like” phenotype have been reported in association with “hypomorphic” mutations in the *CNGA3*, *CNGB3*, *PDE6C*, and *GNAT2* genes.^5^ However, some of these cases arguably have features more in keeping with incomplete achromatopsia per se, rather than oligocone trichromacy.^5^

Electroretinograms according to the protocol recommendations by the International Society for Clinical Electrophysiology of Vision (ISCEV)^8^, ^9^ also do not allow for the distinction to be made between *RGS9*/*R9AP-*associated retinopathy and oligocone trichromacy, with more comprehensive electroretinograms than those mandated by the ISCEV in the electroretinogram standard document being needed. Extended electroretinogram testing includes dark-adapted red flash electroretinogram (which has both a cone and rod system component) and dark-adapted 10.0 (or 11.5) electroretinograms with a wider range of increasing interstimulus intervals are necessary to establish the correct diagnosis.^3^, ^10^ However, both oligocone trichromacy and *RGS9*/*R9AP-*associated retinopathy are associated with generalized cone system dysfunction, with an undetectable light-adapted 30 Hz flicker electroretinogram and a severely reduced light-adapted 3.0 electroretinogram.

Recently, photoreceptor topography was assessed in 3 subjects with typical oligocone trichromacy, confirmed to not harbor variants in either *RGS9* or *R9AP*, using adaptive-optics flood-illuminated ophthalmoscopy (AO-Flood).^11^ These patients were all found to have a reduced number of waveguiding cones at the fovea, with no structure visible outside the central fovea—thereby confirming the original hypothesis of the underlying basis of oligocone trichromacy, that the disorder was caused by a reduction in number of otherwise functional cone photoreceptors.

In this study, we have undertaken deep phenotyping of molecularly proven patients with either *RGS9-* or *R9AP-*associated retinopathy. Spectral-domain optical coherence tomography was used to both qualitatively and quantitatively examine retinal laminar integrity and confocal adaptive-optics scanning light ophthalmoscopy was used to directly probe photoreceptor mosaic architecture in order to determine (1) whether oligocone trichromacy and *RGS9*/*R9AP-*associated retinopathy could be discerned at the cellular level, and (2) whether the generalized retinal dysfunction in *RGS9*/*R9AP-*associated retinopathy is secondary to cone cell loss/structural deficit or a functional deficit in otherwise intact receptors.

## Subjects and Methods

### Subjects

This observational case series was conducted in accordance with the tenets of the Declaration of Helsinki (1983 Revision) and the applicable regulatory requirements, and in compliance with the Health Insurance Portability and Accountability Act (HIPAA). After approval of the study and its procedures by the local ethics committees of Moorfields Eye Hospital and the Medical College of Wisconsin, informed consent was obtained from all participating subjects prior to enrollment.

All 3 subjects have been previously reported: subjects MM_0032, MM_0033, and JC_0759 correspond to cases 1A, 2A, and 3, respectively, in the series published by Michaelides and associates.^3^ The Table summarizes clinical findings and subject demographics. All subjects had the pathognomonic electroretinogram findings associated with bradyopsia as described previously^3^: In brief, the dark-adapted 0.01 electroretinogram (rod electroretinogram), the dark-adapted red flash electroretinogram (which has both a cone and rod system component), and the dark-adapted 3.0 electroretinogram (combined rod-cone standard flash electroretinogram; rod dominated given numerosity) with interstimulus interval of 2 minutes were all normal. However, with an interstimulus interval of 20 seconds, amplitude reduction was observed in DA 11.5 electroretinograms, which was progressively less marked with increasing interstimulus interval, consistent with delayed recovery after the flash. Light-adapted testing revealed an undetectable pattern electroretinogram and 30 Hz flicker electroretinogram.

Pupils of each patient were dilated using 1 drop each of phenylephrine (2.5%) and tropicamide (1%) before imaging in the study herein.

### Spectral-Domain Optical Coherence Tomography

All subjects underwent spectral-domain optical coherence tomography using an Envisu C2300 system (Bioptigen, Morrisville, North Carolina, USA) through the macular region. Macular scans, either 750 A-scans/B-scan and 150 B-scans or 1000 A-scans/B-scan and 100 B-scans, over a nominally 7 × 7 mm area were acquired to locate the center of the fovea. Then, high-density line scans were acquired (1000 A-scans/B-scan), and ImageJ (NIH, Bethesda, Maryland, USA) was used to register up to 100 B-scans and average them to reduce speckle noise.^11^ To correct for retinal magnification, axial length was measured in all subjects (Zeiss IOL Master; Carl Zeiss Meditec, Dublin, California, USA) in order to estimate the lateral scale of each image and to correct the interindividual differences.^12^ In order to analyze the retinal cross-section and determine total retinal thickness, inner retinal thickness, and outer nuclear layer thickness, foveal scans were manually segmented using ImageJ (NIH) and a Matlab (MATLAB; MathWorks, Natick, Massachusetts, USA)-based algorithm.^13^, ^14^

**Figure 1:**
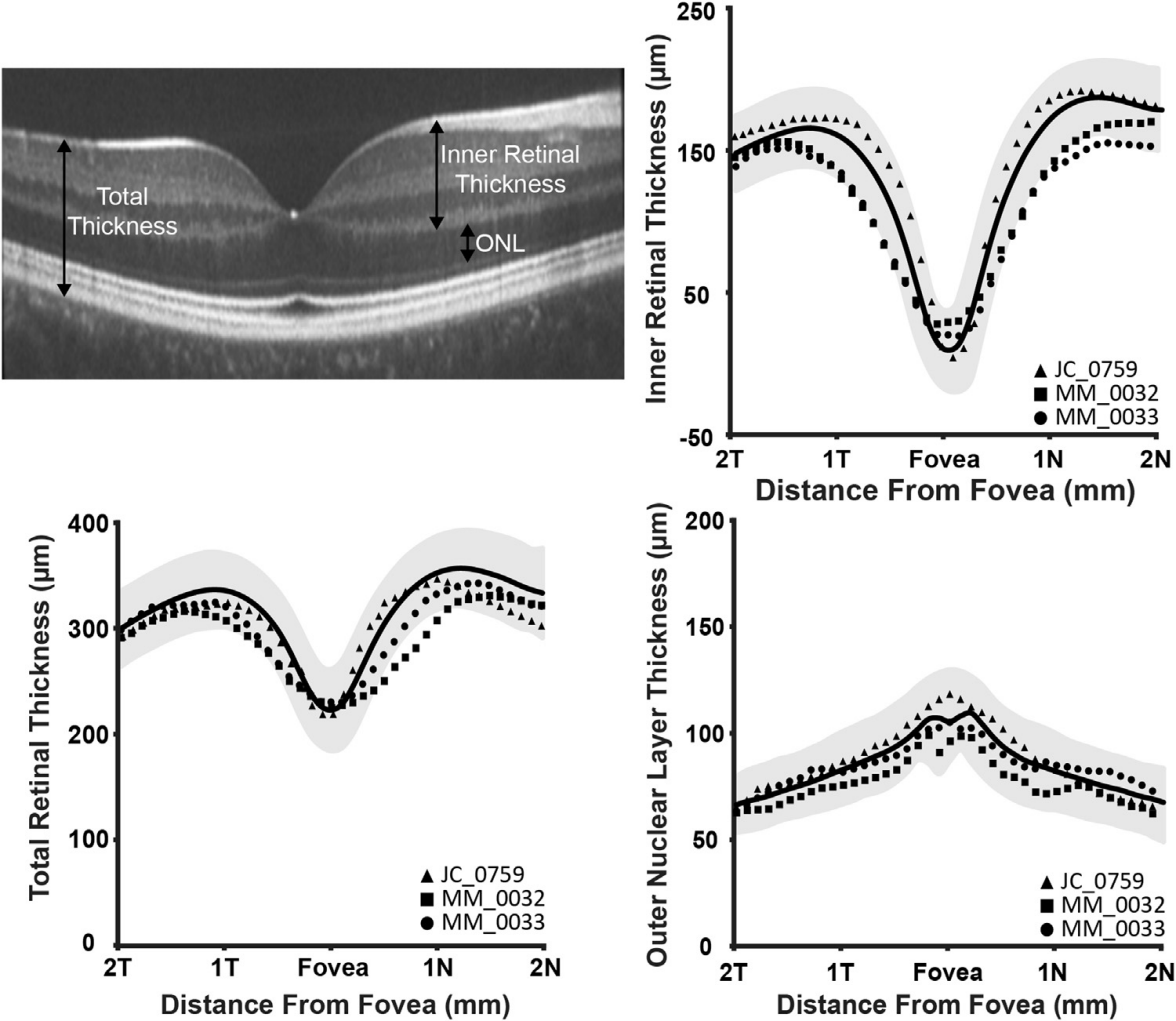
Spectral-domain optical coherence tomography B-scans in 3 subjects with bradyopsia. Top left: A representative macular line scan is shown with designated layer thickness measurements. Bottom left: Total retinal thickness was normal in all subjects compared to a normative database. Normative average thickness is shown as a black line ± 2 standard deviations (gray shaded region). Inner retinal thickness (Top right) and outer nuclear layer thickness (Bottom right) was within the normal range for all subjects compared to a normative database. Subject JC_0759 is represented by a triangle, MM_0032 as a square, and MM_0033 as a circle throughout.

The following definitions were applied: total retinal thickness was defined as the distance from the inner limiting membrane to the retinal pigment epithelium; inner retinal thickness as the distance from the inner limiting membrane to the outer plexiform layer; and outer nuclear layer thickness as the distance from the outer plexiform layer to the external limiting membrane. Layer designations are shown in Figure 1 (Top left). Thicknesses were compared to those derived from a normative database collected at Medical College Wisconsin, consisting of 167 normal individuals (72 male, 95 female, average age of 32.6 years [range 7-60 years]).^13^, ^15^

### Confocal Adaptive-Optics Retinal Imaging

Images of the photoreceptor mosaic were obtained using 1 of 2 nearly identical adaptive-optics scanning light ophthalmoscopes located at Medical College Wisconsin and Moorfields Eye Hospital.^16^, ^17^ The difference between the systems was the center wavelength of the imaging source. The imaging source at Moorfields Eye Hospital was a 790 nm superluminescent diode (Superlum Ireland, Carrigtwohill, County Cork, Ireland) while the imaging source at Medical College Wisconsin was a 775 nm superluminescent diode (Inphenix Inc, Livermore, California, USA). Both systems operated at ∼17 frames/second. Wavefront aberrations were corrected using a 97-actuator deformable mirror (ALPAO, Biviers, France). Image sequences were recorded at different locations across the central fovea and parafovea. Each image sequence was desinusoided and registered using algorithms described by Dubra and associates.^18^ The resultant image from each sequence was then manually combined into a large montage (Adobe Photoshop; Adobe Systems, Inc, San Jose, California, USA). Each subject's axial length was used to determine the absolute scale of his or her retinal images, ensuring the accuracy of subsequent measurements of the cone mosaic.

From these images, cone density measurements were obtained using a Matlab-aided direct counting procedure.^12^, ^13^ Cone photoreceptors were identified within an 80 × 80 μm region of interest at the location of peak cone density (0) when possible, and at 100, 200, 300, 400, 600, 800, 1000, 1200, and 1400 μm temporal eccentricity. Cone density was then calculated over the central 55 × 55 μm area to mitigate the effect of edge artifacts.^19^ Cone densities were compared to normal data published by Curcio and associates.^20^ Owing to the focal foveal lesion present in JC_0759, the center of the lesion was assumed to be the location of peak cone density, and all measurements were based from this location.

## Results

### Spectral-Domain Optical Coherence Tomography

**Figure 2:**
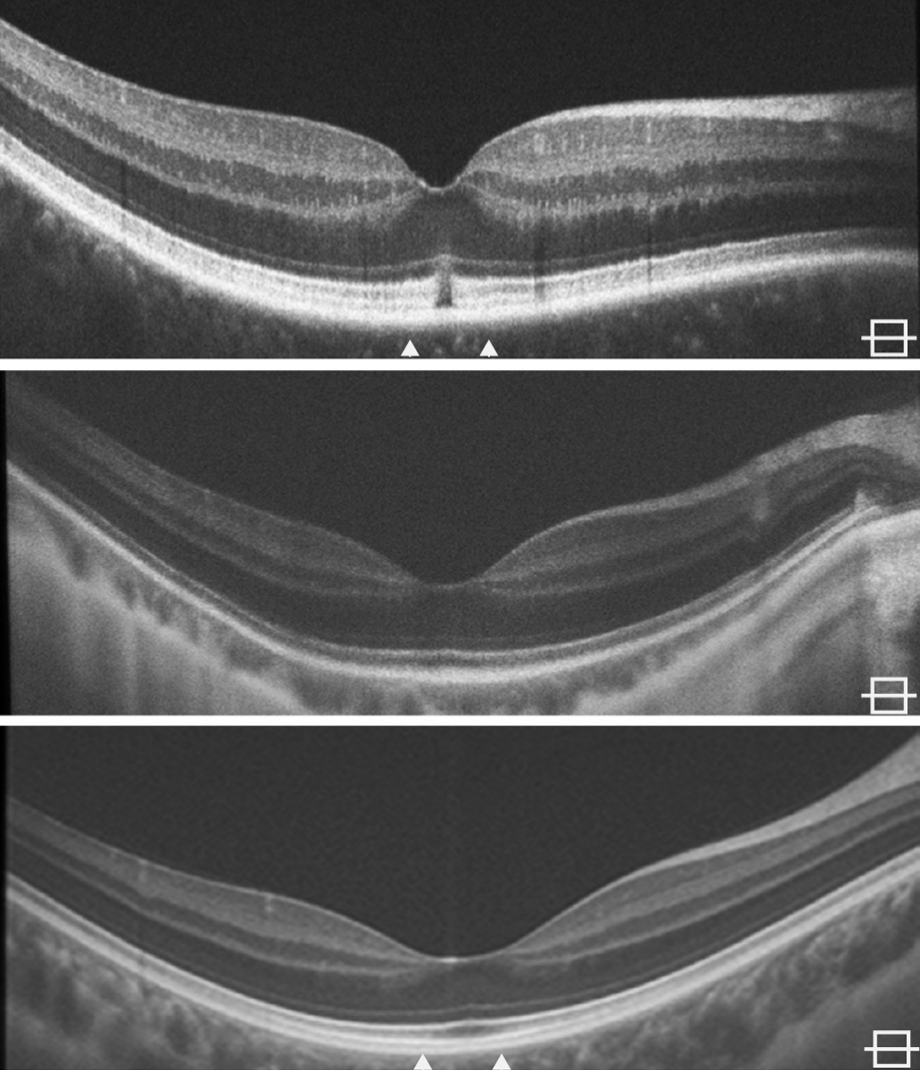
Horizontal spectral-domain optical coherence tomography images through the fovea of all 3 bradyopsia subjects. Qualitative spectral-domain optical coherence tomography analysis shows a focal disruption in the inner segment ellipsoid and interdigitation zone in JC_0759 (Top). This contrasts to the intact outer retinal lamination present in MM_0032 (Middle) and MM_0033 (Bottom). The arrows on JC_0759 and MM_0033 indicate the location of the adaptive-optics scanning light ophthalmoscopy montage shown in Figure 3.

Volumetric and foveal spectral-domain optical coherence tomography images were obtained in both eyes of all subjects. Foveal scans from all subjects are shown in Figure 2. Qualitative assessment of the inner segment ellipsoid band showed that JC_0759 had focal foveal disruption of the inner segment ellipsoid band in both eyes, while there was normal macular lamination in both eyes of MM_0032 and MM_0033 (Figure 2). Total, inner retinal, and outer nuclear layer thickness was within 2 standard deviations of normal for all subjects (Figure 1).

### Qualitative Assessment of the Photoreceptor Mosaic

**Figure 3:**
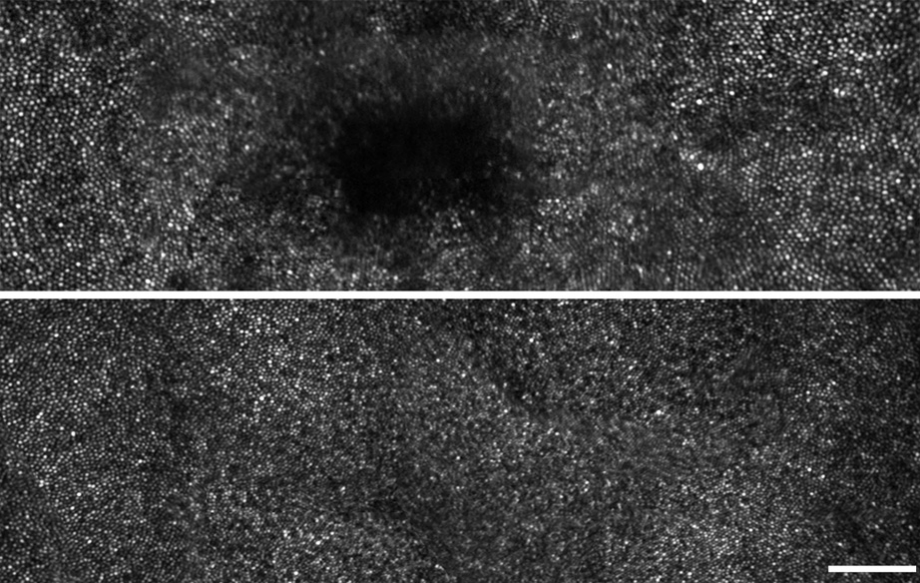
Foveal montages derived from adaptive-optics scanning light ophthalmoscopy for the *RGS9* subject JC_0759 and *R9AP* subject MM_0033. The foveal montage for JC_0759 (Top) shows a hyporeflective lesion that corresponds to the disruption seen on spectral-domain optical coherence tomography in Figure 2. Conversely, the foveal montage from MM_0033 (Bottom) shows a qualitatively normal cone mosaic across the foveal region. Scale bar is 100 μm.

**Figure 4:**
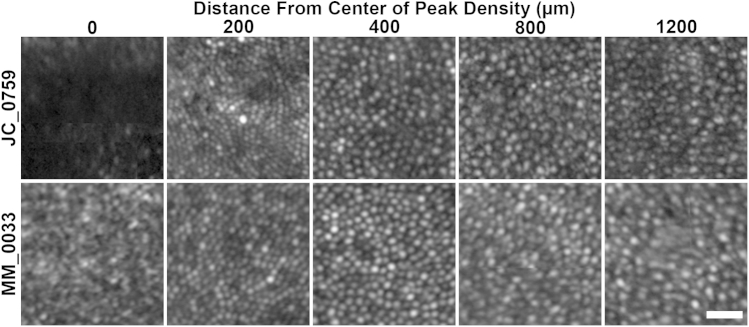
Photoreceptor images from multiple eccentricities obtained with adaptive-optics scanning light ophthalmoscopy in 2 bradyopsia patients. Photoreceptor images are shown at the center of peak density (lesion: JC_0759, Top row, and MM_0033, Bottom row), and 200, 400, 800, and 1200 μm away from center of peak density. Cone photoreceptors were readily identifiable at all locations except the center of JC_0759. Scale bar is 20 μm.

Adaptive-optics scanning light ophthalmoscopy imaging identified a small focal central hyporeflective lesion (nonwaveguiding cones) in both eyes of JC_0759. The lesion was present at the foveal center; however, cone mosaic integrity was intact at all other observed eccentricities. A foveal montage for subject JC_0759's right eye is shown in Figure 3. Subjects MM_0032 and MM_0033 had qualitatively normal waveguiding cone photoreceptors at all imaged locations. The foveal montage for subject MM_0033's right eye is shown in Figure 3. Selected location photoreceptor images are shown in Figure 4. Consistent with the focal lesion in JC_0759, no photoreceptors are visible at the center using confocal adaptive-optics scanning light ophthalmoscopy. Cone photoreceptors are visible (roughly equally sized circular objects) in the images from peak density until 400 μm from the center.

### Quantitative Assessment of Cone Photoreceptor Density

**Figure 5:**
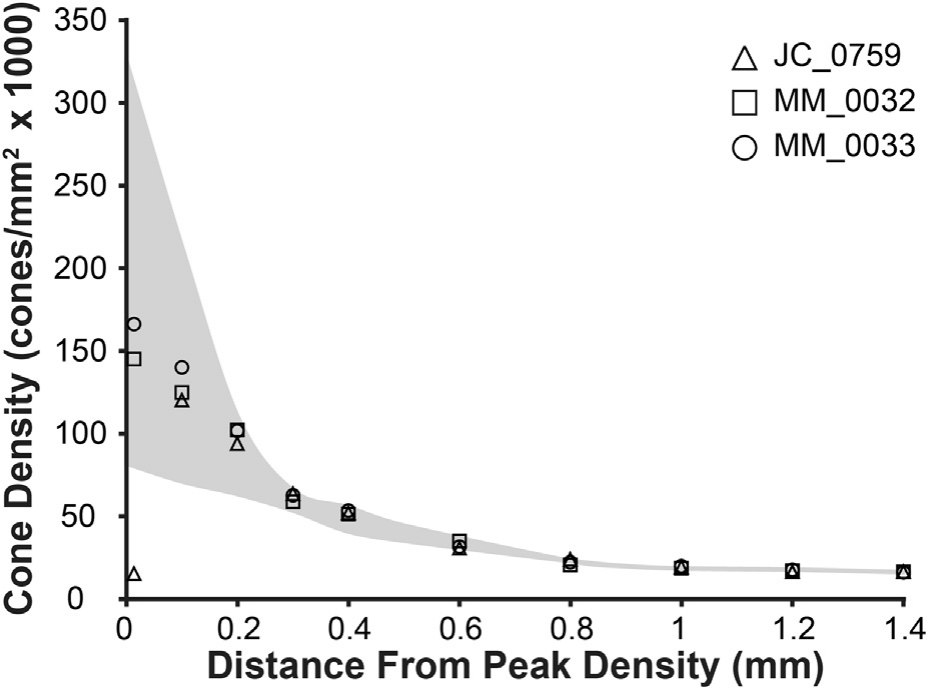
Cone photoreceptor density in relation to eccentricity in 3 subjects with bradyopsia. Normal cone density established by histologic analysis (Curcio and associates^20^) is shown as the gray shaded region. Subject JC_0759 is represented by a triangle, MM_0032 as a square, and MM_0033 as a circle. The 3 subjects with bradyopsia have very similar cone densities from 0.3 mm from peak density and thereby their respective symbols overlap.

Measured cone densities were within the normal range reported in histologic analysis at all 10 locations in both eyes of subjects MM_0032 and MM_0033.^20^ The aforementioned foveal defect in patient JC_0759 was reflected by a decreased cell number at the area of expected peak cone density. Cone density at 100 μm from center, the location closest to center measureable for all 3 subjects, ranged from 123 132 to 140 013 cones/mm^2^. Cone density ranged from 30 573 to 34 876 cones/mm^2^ by 600 μm from center and 15 987 to 16 253 cones/mm^2^ by 1400 μm. These cone densities at different locations are entirely in keeping with both published histologic and in vivo image analysis.^20^, ^21^, ^22^ Quantification of cone photoreceptor density at all measured eccentricities is shown in Figure 5.

## Discussion

Oligocone trichromacy and *RGS9*/*R9AP-*associated retinopathy share clinical characteristics including stationary cone dysfunction, mild photophobia, normal color vision, and normal fundi. *RGS9*/*R9AP-*associated retinopathy can be distinguished from oligocone trichromacy on the basis of molecular screening and distinct electrophysiologic findings following extended assessment.^3^, ^5^, ^10^ We conducted this study to characterize the retinal architecture in *RGS9*/*R9AP-*associated retinopathy and thereby shed light into any structural differences between oligocone trichromacy and *RGS9*/*R9AP-*associated retinopathy. We hypothesized that they could be discerned on the basis of their cellular phenotype.

**Figure 6:**
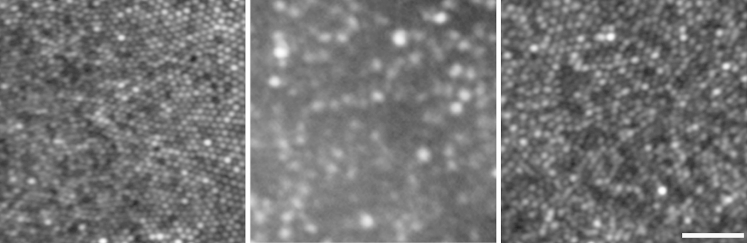
Comparison of foveal cone mosaics as imaged by adaptive-optics light ophthaloscopy in normal subjects and subjects with oligocone trichromacy and *RGS9*/*R9AP-*associated retinopathy. Normal (Left) and *RGS9*/*R9AP-*associated retinopathy (Right) subjects have a continuous cone mosaic across the foveal region. In contrast, a decreased cone mosaic is observed in oligocone trichromacy (Middle). Scale bar is 25 μm.

In a previous study in oligocone trichromacy, cone photoreceptor imaging identified 2 types of subject. In 3 (out of 4) subjects, representing typical oligocone trichromacy, a uniform reduction in cone density was identified, in combination with a reduced outer nuclear layer thickness on spectral-domain optical coherence tomography; these findings support the original hypothesis that oligocone trichromacy is characterized by a reduced number of functional cones.^11^ The findings of our present study in *RGS9*/*R9AP-*associated retinopathy are in direct contrast, providing evidence that cones are present in normal numbers but are dysfunctional (Figure 6). This is in keeping with the normal cone density identified histopathologically in *RGS9*-knockout mice.^23^

Adaptive-optics scanning light ophthalmoscopy imaging revealed a small focal hyporeflective lesion in both eyes at the foveal center in the subject with *RGS9*-associated retinopathy, which also correlated with a decreased cell number at this location, as well as bilateral focal interruption of the interdigitation zone and inner segment ellipsoid bands on spectral-domain optical coherence tomography. There are at least 3 possible explanations for these structural changes: (1) they may be directly associated with the *RGS9* genotype; (2) they may be age related (indicative of progression), given that the subject with *RGS9* variants is significantly older than the *R9AP* subjects; and (3) since the *RGS9* subject also reported repeated blunt trauma to both eyes in the past, the possibility of an unrelated comorbidity cannot be entirely ruled out, especially given previous reports of subclincial photoreceptor disruption in response to trauma.^24^, ^25^ High-resolution imaging of further molecularly proven patients, perhaps using newer nonconfocal imaging,^26^ will help to clarify the underlying cause of the outer lamellar defect and photoreceptor mosaic disruption.

Both RGS9 and R9AP play critical roles in enabling the rapid recovery of the phototransduction cascade.^3^ RGS9 significantly accelerates the hydrolysis of α-transducin bound guanosine-5′-triphosphate to guanosine diphosphate, with lack of RGS9 leading to a substantial delay in the recovery from light response in knockout mice.^2^, ^27^ Similar observations have been made in the absence of R9AP, which serves as an anchor protein for RGS9 to the photoreceptor outer segment membrane and enhances its activity by up to 70-fold.^2^, ^28^ This delayed recovery phase is reflected in subjects with *RGS9*/*R9AP-*associated retinopathy by electrophysiologic assessment; with the dark-adapted 10.0 (or 11.5) electroretinograms with ISCEV standard interstimulus interval of 20 seconds showing amplitude reduction, which is progressively less severe with increasing interstimulus interval, consistent with delayed recovery following the flash, thereby demonstrating the need for an extended interstimulus interval to obtain full recovery of the electroretinogram following the previous flash.^3^, ^10^

In *RGS9*/*R9AP-*associated retinopathy subjects, the dark-adapted 0.01 electroretinogram (rod electroretinogram), the dark-adapted red flash electroretinogram (both an early cone and later rod system component), and the dark-adapted 3.0 electroretinogram (combined rod-cone standard flash electroretinogram; rod dominated given numerosity) with interstimulus interval of 2 minutes are all normal—all in keeping with the relatively intact cone and rod photoreceptor mosaic observed in our study. However, with light-adapted testing, a generalized reduction or absence of cone responses is observed (pattern electroretinogram, light-adapted 30 Hz flicker and light-adapted 3.0 electroretinograms). This may at first sight appear to be incongruous with structural observations. However, aforementioned electroretinograms to a red flash under dark adaptation, which in a normal subject gives an early cone system–derived response and a later rod system–derived response, are completely normal, showing that dark-adapted cones function normally, at least initially. Indeed, even dark-adapted 30 Hz flicker responses (albeit to a dim flash, with a short presentation time) are normal initially, but become undetectable after approximately 10 seconds of stimulation.^3^, ^10^

These observations thereby are all in keeping with our findings that cones are not only present in normal density, but also capable of normal function, and thus potentially amenable to rescue. While patients with either *RGS9*/*R9AP-*associated retinopathy or oligocone trichromacy have very similar clinical phenotypes, we highlight the utility of cellular imaging in both effectively distinguishing between these conditions and determining the potential for therapeutic intervention. Adaptive-optics scanning light ophthalmoscopy will be valuable to assess whether *RGS9*/*R9AP-*associated retinopathy is indeed an entirely stationary condition, given our findings in the *RGS9* subject and the increasing evidence of progression in the cone dysfunction syndromes.^5^, ^14^

## Figures and Tables

**Table:** Demographics and Clinical Findings for 3 Subjects With Genetically Confirmed Bradyopsia

Subject Number	Age (y)	Sex	Visual Acuity		Axial Length (mm)		Gene	Allele 1 / Allele 2	AOSLO Used
OD	OS	OD	OS
JC_0759	62	M	6/12	6/12	23.16	22.92	*RGS9*	p.W299R / p.R128X	MCW
MM_0032	25	F	6/12	6/12	30.36	27.84	*R9AP*	p.D32_Q34del / p.D32_Q34del	MEH
MM_0033	16	F	6/18	6/12	24.98	24.84	*R9AP*	p.D32_Q34del / p.D32_Q34del	MEH

AOSLO = adaptive-optics scanning light ophthalmoscope; MCW = Medical College of Wisconsin; MEH = Moorfields Eye Hospital.
